# Correction: Factors affecting antenatal corticosteroid use in low- and middle-income countries: Facility characteristics, structural readiness, and past performance of CEmONC signal functions

**DOI:** 10.1371/journal.pgph.0006470

**Published:** 2026-05-12

**Authors:** Wen-Chien Yang, Catherine Arsenault, Victoria Y. Fan, Nazia Binte Ali, Fadhlun Mohamed Alwy Al-beity, Emily R. Smith

There are errors in the Funding statement. The correct Funding statement is as follows: This work was supported by a Research Enhancement Award from the Office of Research Excellence, Milken Institute School of Public Health, George Washington University, USA.

In the methods section of the abstract, there is an error in the third sentence. The correct sentence should read: We conducted mixed-effect logistic regressions.

In the results section of the abstract, there are errors starting from the third sentence. The correct sentences should read: Urban facilities had 33% higher odds of recent ACS use (95% CI 9%–61%) than rural facilities. Corticosteroid availability was associated with 21% higher odds of recent ACS use (95% CI 2%–44%). Facilities in the highest readiness tertile were more likely to have recent ACS use than those in the lowest (OR 2.58, 95% CI 1.99–3.34). Each CEmONC signal function, except assisted vaginal deliveries, was significantly associated with recent ACS use, with neonatal resuscitation having the largest effect (OR 3.17, 95% CI 2.23–4.49).

In the methods section, there is an error in the fifth sentence under the heading of statistical analysis. The correct sentence should read: We then used mixed-effect logistic regressions to report adjusted odds ratios (OR).

In the results section, there are errors in the first two sentences and in the fourth through the seventh sentences under the heading of Factors affecting recent ACS use. The correct first two sentences should read: Table 2 details the results of mixed-effect logistic regressions, showing that facility characteristics, structural readiness, and past performance of CEmONC are associated with recent ACS use. Urban facilities had 33% higher odds of recent ACS use than rural facilities (OR 1.33, 95% CI 1.09–1.61). The correct fourth through seventh sentences should read: Compared to facilities without injectable corticosteroids or ultrasound, the availability of injectable corticosteroids and ultrasound were associated with increased odds of recent ACS use by 21% (OR 1.21, 95% CI 1.02–1.44) and 43% (OR 1.43, 95% CI 1.15–1.76), respectively. Facilities in the highest readiness tertile had higher odds of recent ACS use than those in the lowest (OR 2.58, 95% CI 1.99–3.34). Staffing types affected recent ACS use: facilities with at least one medical doctor, midwife, or specialist had increased odds of recent ACS use by 50% (OR 1.50, 95% CI 1.22–1.85), 36% (OR 1.36, 95% CI 1.11–1.67), and 42% (OR 1.42, 95% CI 1.13–1.78), respectively. Lastly, past performance of each CEmONC signal function, except for assisted vaginal deliveries, was significantly associated with more recent ACS use, with performing neonatal resuscitation having the largest effect (OR 3.17, 95% CI 2.23–4.49).

In the discussion section, there are errors in the seventh sentence of the fourth paragraph. The correct sentence should read: The effect sizes for some CEmONC signal functions (OR 3.17 for neonatal resuscitation, OR 2.22 for performing manual removal of placenta, and OR 2.29 for performing Cesarean sections) are much larger than that for corticosteroid availability (OR 1.21).

There are errors in Table 2. Please see the correct Table 2 below.

There are errors in S5 Table. Please see the correct S5 Table below.

**Table 2:** Mixed effect logistic regression models of recent antenatal corticosteroid utilization.

	Proportion of facilities with recent ACS use (%)^1^	Bivariate regrssion^2^			Multivariate regression^3^		
**odds ratio** **(OR)**	**95% CI**	**p-value**	**Adjusted** **odds ratio (aOR)**	**95% CI**	**p-value**
**Country**							
Afghanistan 2018–2019	59.4%	**2.89**	**1.45 – 5.78**	**0.003**	**0.70**	**0.31 – 1.60**	**0.399**
Nepal 2021	8.2%	**0.39**	**0.22 – 0.69**	0.001	**0.19**	**0.10 – 0.36**	<0.001
Haiti 2017–2018	23.6%	**0.53**	**0.30 – 0.92**	**0.025**	**0.17**	**0.09 – 0.34**	<0.001
DRC 2017–2018	22.2%	**1.07**	**0.69 – 1.67**	**0.756**	**0.70**	**0.41 – 1.19**	**0.187**
Ethiopia 2021–2022	27.4%	**1.78**	**1.06 – 3.00**	**0.029**	**0.83**	**0.46 – 1.52**	**0.552**
Malawi 2013–2014	21.5%	**0.37**	**0.20 – 0.69**	0.002	**0.41**	**0.20 – 0.85**	**0.016**
Senegal 2018 and 2019	23.4%	**0.61**	**0.37 – 1.00**	**0.051**	**0.80**	**0.44 – 1.45**	**0.460**
Tanzania 2014–2015	4.0%	**0.30**	**0.19 – 0.47**	<0.001	**0.14**	**0.08 – 0.24**	<0.001
Bangladesh 2017–2018^4^	23.2%	*ref*			*ref*		
**Facility characteristics**							
*Location*							
Urban *versus* rural^5^	44.5% *versus* 21.1%	**4.62**	**3.99 – 5.36**	**<0.001**	**1.33**	**1.09 – 1.61**	**0.005**
*Managing authority type*							
Public	28.0%	*ref*			*ref*		
Private for-profit	43.0%	**1.76**	**1.47 – 2.12**	**<0.001**	**0.70**	**0.55 – 0.90**	**0.006**
Private not-for-profit/faith or mission-based	37.8%	**1.67**	**1.40 – 1.99**	**<0.001**	**0.79**	**0.63 – 0.99**	**0.039**
Others	19.5%	**1.04**	**0.58 – 1.86**	**0.904**	**0.98**	**0.50 – 1.94**	**0.962**
**Structural readiness**							
Corticosteroid availability *versus* unavailability^6^	45.1% *versus* 18.9%	**3.46**	**3.02 – 3.96**	**<0.001**	**1.21**	**1.02 – 1.44**	**0.030**
Ultrasound availability *versus* unavailability^6^	60.9% *versus* 19.3%	**8.78**	**7.54 –10.23**	**<0.001**	**1.43**	**1.15 – 1.76**	0.001
*Readiness tertile* ^7^							
High	48.0%	**15.35**	**12.35 –19.08**	**<0.001**	**2.58**	**1.99 – 3.34**	<0.001
Middle	21.9%	**2.76**	**2.24 – 3.39**	**<0.001**	**1.30**	**1.03 – 1.64**	**0.028**
Low	14.6%	*ref*			*ref*		
*Staffing* ^8^							
At least one medical doctor *versus* no medical doctor^9^	56.5% *versus* 13.2%	**9.60**	**8.27 – 11.13**	**<0.001**	**1.50**	**1.22 – 1.85**	<0.001
At least one midwife *versus* no midwife^9^	38.8% *versus* 25.1%	**5.18**	**4.39 – 6.13**	**<0.001**	**1.36**	**1.11 – 1.67**	**0.003**
At least one specialist *versus* no specialist^9^	61.8% *versus* 23.4%	**7.80**	**6.59 – 9.23**	**<0.001**	**1.42**	**1.13 – 1.78**	**0.002**
**CEmONC signal functions** ^10^							
Ever provide parenteral antibiotics *versus* never	37.8% *versus* 6.1%	**9.47**	**7.39 – 12.14**	**<0.001**	**1.49**	**1.10 – 2.01**	**0.009**
Ever provide parenteral oxytocin *versus* never	33.1% *versus* 5.5%	**11.98**	**7.76 – 18.49**	**<0.001**	**1.85**	**1.08 – 3.16**	**0.025**
Ever provide parenteral anticonvulsants *versus* never	46.2% *versus* 11.5%	**7.89**	**6.76 – 9.21**	**<0.001**	**1.91**	**1.57 – 2.31**	<0.001
Ever perform assisted vaginal delivery *versus* never	37.1% *versus* 12.6%	**4.38**	**3.62 – 5.30**	**<0.001**	**1.13**	**0.89 – 1.44**	**0.319**
Ever perform manual removal of placenta *versus* never	38.2% *versus* 7.2%	**7.13**	**5.71 – 8.91**	**<0.001**	**2.22**	**1.69 – 2.91**	<0.001
Ever perform removal of retained products *versus* never	39.8% *versus* 11.8%	**4.84**	**4.11 – 5.70**	**<0.001**	**1.51**	**1.23 – 1.87**	<0.001
Ever perform neonatal resuscitation *versus* never	36.7% *versus* 4.4%	**12.45**	**9.18 – 16.89**	**<0.001**	**3.17**	**2.23 – 4.49**	<0.001
Ever perform Cesarean sections *versus* never	63.5% *versus* 13.1%	**15.11**	**12.89 – 17.70**	**<0.001**	**2.29**	**1.75 – 2.99**	<0.001
Ever provide blood transfusion *versus* never	63.8% *versus* 15.1%	**12.31**	**10.58 – 14.32**	**<0.001**	**1.61**	**1.25 – 2.06**	<0.001
**Random effects**							
N of region					113		
τ00 region					**0.27**		
ICC^11^					**0.08**		
Observations (N)					6111		
Marginal R^2^/Conditional R^2^					**0.584/0.615**		

^1^ Recent ACS use was defined as using ACS within the past 3 months. For each country, the proportions were calculated considering facility sampling weight.

^2^ Odds ratios (OR) were derived from bivariate regressions that included each independent variable separately, country fixed effects, and sub-national divisions as random intercepts.

^3^ Adjusted odds ratios (aOR) were derived from mixed effect logistic regression model that included all independent variables, country fixed effects, and sub-national regions as random intercepts.

^4^ Bangladesh was selected as the reference level because the directions of **odds** ratios for other countries remained the same, facilitating a clearer interpretation and understanding of the effects.

^5^ Rural is the reference level.

^6^ Unavailability is the reference level.

^7^ Readiness tertiles refer to country-specific readiness tertiles, calculated by the available numbers of equipment, diagnostics, medicines and commodities, and guidelines.

^8^ These binary variables indicate facilities having at least one medical doctor, midwife, or specialist, which were constructed based on the surveyed staff types for each SPA.

^9^ No medical doctor, no midwife, or no specialist is the reference level.

^10^ Facilities that never performed each CEmONC signal function were viewed as the reference level

^11^ Intraclass correlation coefficient

## Supporting information

S5 Table Mixed effect logistic regression models of recent antenatal corticosteroid utilization among eight countries (excluding Afghanistan). (DOCX)
