# Supplementary material for: Factors affecting antenatal corticosteroid use in low- and middle-income countries: Facility characteristics, structural readiness, and past performance of CEmONC signal functions
Source: PLOS Glob Public Health. 2025 Aug 14;5(8):e0003989. doi: 10.1371/journal.pgph.0003989 (PMC12352826; doi:10.1371/journal.pgph.0003989)
Supplement: S1 Table — (DOCX) [file pgph.0003989.s001.docx]

**S1 Table.** Development of standardized categorization of facility managing authority types

| **Standardized categories of managing authority types^1^** | **Country-specific categorization for managing authority types^2^** | | | | | | | | |
| --- | --- | --- | --- | --- | --- | --- | --- | --- | --- |
|  | Afghanistan  2018-2019 | Nepal  2021 | Haiti  2017 | Malawi  2013-2014 | Tanzania  2014-2015 | Ethiopia  2021-2022 | Bangladesh  2017-2018 | DRC  2017-2018 | Senegal  2018 & 2019 |
| **1) Public** | public/government | public/government | public/government | public/government | Public/government | public/government,  other government (military, federal, etc.) | public/government,  local government | public/government | public/government |
| **2) Private for-profit** | private for-profit | private for-profit | private for-profit | private for-profit | private for-profit | private for-profit | private for-profit | private for-profit | private for-profit |
| **3) Private not-for-profit/mission or faith-based** | private not-for-profit | private not-for-profit,  mission-based facilities | private not-for-profit | private not-for-profit,  mission-based facilities | mission-based facilities | private not-for-profit,  mission or faith-based | private not-for-profit | private not-for-profit,  mission-based facilities | private not-for-profit |
| **4) Others** |  | - | mixed | Christian Health Association of Malawi,  Company | parastatal | - | - | - | - |

^1^We developed the standardized categorization for managing authority types across nine countries.

^2^The country-specific categorizations were obtained from surveys of each country.
