# Supplementary material for: Factors affecting antenatal corticosteroid use in low- and middle-income countries: Facility characteristics, structural readiness, and past performance of CEmONC signal functions
Source: PLOS Glob Public Health. 2025 Aug 14;5(8):e0003989. doi: 10.1371/journal.pgph.0003989 (PMC12352826; doi:10.1371/journal.pgph.0003989)
Supplement: S2 Table — (DOCX) [file pgph.0003989.s002.docx]

**S2 Table.** Items included to develop structural readiness tertiles

| **Readiness items included (number of items = 28)**^1^ |
| --- |
| **Equipment (n=16)**  thermometer, delivery pack, cord clamp, manual vacuum extractor, vacuum aspiratory kit or D&C kit, forceps (large), forceps (middle), suction bulb or penguin sucker, stethoscope, newborn masks and neonatal size self-inflating bag, incubator, other external heat source, pulse oximeter, oxygen concentrator, filled oxygen cylinder, oxygen distribution system  **Diagnostics (n=3)**  hematology analyzer, HIV rapid diagnostic test, syphilis rapid diagnostic test  **Medicines and commodities (n=7)**  parenteral antibiotics, parenteral oxytocin, parenteral anticonvulsant, glucometer, glucometer strips, hand-washing soap, disposable latex gloves  **Guidelines (n=2)**  national guideline for CEmONC^2^, guideline on management of preterm labor |

^1^ Not all surveys included in this study surveyed all these 28 items. Bangladesh 2017-2018 did not survey HIV diagnostic test; Nepal 2021 did not survey HIV diagnostic test, CEmONC, and guidelines on the management of preterm labor; DRC 2017-2018 did not survey oxygen distribution system. Readiness tertiles were constructed based on the country-specific number of items surveyed.

^2^ CEmONC: Comprehensive Emergency Obstetric and Newborn Care
