# Supplementary material for: Factors affecting antenatal corticosteroid use in low- and middle-income countries: Facility characteristics, structural readiness, and past performance of CEmONC signal functions
Source: PLOS Glob Public Health. 2025 Aug 14;5(8):e0003989. doi: 10.1371/journal.pgph.0003989 (PMC12352826; doi:10.1371/journal.pgph.0003989)
Supplement: S3 Table — (DOCX) [file pgph.0003989.s003.docx]

**S3 Table.** Types of staff used to construct binary variables indicating at least one medical doctor, midwife, obstetrician/gynecologist, or specialist

|  | **Types of staff included to construct binary variables** | | | | |
| --- | --- | --- | --- | --- | --- |
|  | **At least one medical doctor** | **At least one midwife** | **At least one obstetrician/gynecologist** | **At least one pediatrician** | **At least one specialist** |
| Afghanistan 2018-2019 | general medical doctor | registered midwife, community midwife | NA^1^ | NA^1^ | specialist |
| Bangladesh 2017-2018 | senior consultant (medicine), junior consultant (medicine), residential medical officer (RMO), medical officer (MO)/physician, indoor medical officer (IMO), indoor medical officer (IMO) medicine | nurse midwife, midwife | senior consultant (obgyn), junior consultant (obgyn),  indoor medical officer (IMO) obgyn | senior consultant (pediatrician), junior consultant (pediatrician),  indoor medical officer (IMO) pediatrician | specialist^2^ |
| Nepal 2021 | generalist | nurse (MN, BSc nurse, BN, PCL, midwife) | gynecologist/obstetrician | pediatrician | specialist^2^ |
| Haiti 2017-2018 | generalist | nurse (health nurse, community nurse, midwife) | NA^1^ | NA^1^ | specialist |
| DRC 2017-2018 | generalist | midwife | NA^1^ | NA^1^ | specialist |
| Ethiopia 2021-2022 | generalist | midwife (BSc), midwife (diploma) | gynecologist/obstetrician | pediatrician | specialist^2^ |
| Malawi 2013-2014 | generalist | registered nurse midwife (BSc), enrolled midwife/nurse midwife technician, enrolled nurse midwife | NA^1^ | NA^1^ | specialist |
| Senegal 2018 and 2019 | generalist | midwife | NA^1^ | NA^1^ | specialist |
| Tanzania 2014-2015 | generalist | registered nurse (including nursing officers and midwives) | NA^1^ | NA^1^ | specialist |

^1^ Afghanistan, Haiti, DRC, Malawi, Senegal, and Ethiopia did not specifically survey the availability of obstetrician/gynecologist and pediatrician; they surveyed the availability of specialists.

^2^ For Bangladesh, Nepal, and Ethiopia, the variable of at least one specialist was constructed based on the variables of at least one obstetrician/gynecologist and at least one pediatrician.
