# Supplementary material for: Factors affecting antenatal corticosteroid use in low- and middle-income countries: Facility characteristics, structural readiness, and past performance of CEmONC signal functions
Source: PLOS Glob Public Health. 2025 Aug 14;5(8):e0003989. doi: 10.1371/journal.pgph.0003989 (PMC12352826; doi:10.1371/journal.pgph.0003989)
Supplement: S4 Table — (DOCX) [file pgph.0003989.s004.docx]

**S4 Table**. Facility distribution across readiness tertiles by managing authority type*

|  | **Afghanistan** | | | **Bangladesh** | | | **Nepal** | | |
| --- | --- | --- | --- | --- | --- | --- | --- | --- | --- |
| **Readiness tertile**  **Managing**  **authority type** | High | Middle | Low | High | Middle | Low | High | Middle | Low |
| Public | 9 / 14 (64.3%) | 3 / 14 (21.4%) | 2 / 14 (14.3%) | 353 / 682 (51.8%) | 315 / 682 (46.2%) | 14 / 682 (2.1%) | 364 / 631 (57.7%) | 267 / 631 (42.3%) | 0 / 631 (0%) |
| Private-for-profit | 21 / 71 (29.6%) | 19 / 71 (26.8%) | 31 / 71 (43.7%) | 97 / 104 (93.3%) | 6 / 104 (5.8%) | 1 / 104 (1.0%) | 112 / 152 (73.7%) | 37 / 152 (24.3%) | 3 / 152 (2.0%) |
| Private-not-for-profit | 5 / 16 (31.2%) | 8 / 16 (50%) | 3 / 16 (18.8%) | 32 / 36 (88.9%) | 4 / 36 (11.1%) | 0 / 36 (0%) | 21 / 22 (95.5%) | 1 / 22 (4.5%) | 0 / 22 (0%) |
| Others | NA | NA | NA | NA | NA | NA | NA | NA | NA |

|  | **Haiti** | | | **DRC** | | | **Ethiopia** | | |
| --- | --- | --- | --- | --- | --- | --- | --- | --- | --- |
| **Readiness tertile**  **Managing**  **authority type** | High | Middle | Low | High | Middle | Low | High | Middle | Low |
| Public | 116 / 156 (74.4%) | 39 / 156 (25%) | 1 / 156 (0.6%) | 164 / 823 (19.9%) | 261 / 823 (31.7%) | 398 / 823 (48.4%) | 151 / 555 (27.2%) | 231 / 555 (41.6%) | 173 / 555 (31.2%) |
| Private-for-profit | 77 / 89 (86.5%) | 10 / 89 (11.2%) | 2 / 89 (2.2%) | 46 / 153 (30.1%) | 51 / 153 (33.3%) | 56 / 153 (36.6%) | 33 / 75 (44.0%) | 29 / 75 (38.7%) | 13 / 75 (17.3%) |
| Private-not-for-profit | 40 / 52 (76.9%) | 12 / 52 (23.1%) | 0 / 52 (0%) | 171 / 377 (45.4%) | 112 / 377 (29.7%) | 94 / 377 (24.9%) | 6 / 15 (40.0%) | 7 / 15 (46.7%) | 2 / 15 (13.3%) |
| Others | 53 / 65 (81.5%) | 10 / 65 (15.4%) | 2 / 65 (3.1%) | NA | NA | NA | NA | NA | NA |

|  | **Malawi** | | | **Senegal** | | | **Tanzania** | | |
| --- | --- | --- | --- | --- | --- | --- | --- | --- | --- |
| **Readiness tertile**  **Managing**  **authority type** | High | Middle | Low | High | Middle | Low | High | Middle | Low |
| Public | 113 / 356 (31.7%) | 143 / 356 (40.2%) | 100 / 356 (28.1%) | 148 / 537 (27.6%) | 227 / 537 (42.3%) | 162 / 537 (30.2%) | 165 / 692 (23.8%) | 308 / 692 (44.5%) | 219 / 692 (31.6%) |
| Private-for-profit | 12 / 25 (48.0%) | 3 / 25 (12.0%) | 10 / 25 (40.0%) | 19 / 37 (51.4%) | 7 / 37 (18.9%) | 11 / 37 (29.7%) | 34 / 70 (48.6%) | 28 / 70 (40.0%) | 8 / 70 (11.4%) |
| Private-not-for-profit | 72 / 148 (48.6%) | 48 / 148 (32.4%) | 28 / 148 (18.9%) | 6 / 19 (31.6%) | 10 / 19 (52.6%) | 3 / 19 (15.8%) | 113 / 180 (62.8%) | 47 / 180 (26.1%) | 20 / 180 (11.1%) |
| Others | 4 / 13 (30.8%) | 7 / 13 (53.8%) | 2 / 13 (15.4%) | NA | NA | NA | 8 / 10 (80%) | 1 / 10 (10%) | 1 / 10 (10%) |

* Presented the proportions of readiness tertiles within each managing authority type
