# Supplementary material for: Factors affecting antenatal corticosteroid use in low- and middle-income countries: Facility characteristics, structural readiness, and past performance of CEmONC signal functions
Source: PLOS Glob Public Health. 2025 Aug 14;5(8):e0003989. doi: 10.1371/journal.pgph.0003989 (PMC12352826; doi:10.1371/journal.pgph.0003989)
Supplement: S5 Table — (DOCX) [file pgph.0003989.s005.docx]

**S5 Table.** Mixed effect log binomial regression models of recent antenatal corticosteroid utilization among eight countries (excluding Afghanistan)

|  | **Proportion of facilities with recent ACS use (%)**^1^ | **Bivariate regrssion**^2^ | | | **Multivariate regression**^3^ | | |
| --- | --- | --- | --- | --- | --- | --- | --- |
|  |  | **relative risk**  **(RR)** | **95% CI** | **p-value** | **Adjusted**  **relative risk (aRR)** | **95% CI** | **p-value** |
| **Country** |  |  |  |  |  |  |  |
| Nepal 2021 | 8.2% | 0.45 | 0.28 – 0.71 | **0.001** | 0.34 | 0.22 – 0.51 | **<0.001** |
| Haiti 2017-2018 | 23.6% | 0.58 | 0.37 – 0.93 | **0.024** | 0.32 | 0.21 – 0.50 | **<0.001** |
| DRC 2017-2018 | 22.2% | 1.05 | 0.73 – 1.50 | 0.803 | 0.76 | 0.54 – 1.08 | 0.129 |
| Ethiopia 2021-2022 | 27.4% | 1.53 | 1.01 – 2.32 | **0.046** | 0.81 | 0.55 – 1.19 | 0.276 |
| Malawi 2013-2014 | 21.5% | 0.44 | 0.26 – 0.73 | **0.002** | 0.54 | 0.33 – 0.88 | **0.013** |
| Senegal 2018 and 2019 | 23.4% | 0.66 | 0.44 – 1.00 | **0.049** | 0.95 | 0.64 – 1.43 | 0.816 |
| Tanzania 2014-2015 | 4.0% | 0.35 | 0.24 – 0.51 | **<0.001** | 0.25 | 0.17 – 0.36 | **<0.001** |
| Bangladesh 2017-2018^4^ | 23.2% | *ref* |  |  | *ref* |  |  |
| **Facility characteristics** |  |  |  |  |  |  |  |
| *Location* |  |  |  |  |  |  |  |
| Urban *versus* rural^5^ | 44.5% *versus* 21.1% | 3.09 | 2.77 – 3.45 | **<0.001** | 1.21 | 1.06 – 1.39 | **0.004** |
| *Managing authority type* |  |  |  |  |  |  |  |
| Public | 28.0% | *ref* |  |  | *Ref* |  |  |
| Private for-profit | 43.0% | 1.56 | 1.36 – 1.80 | **<0.001** | 0.78 | 0.66 – 0.92 | **0.003** |
| Private not-for-profit/faith or mission-based | 37.8% | 1.51 | 1.31 – 1.73 | **<0.001** | 0.86 | 0.73 – 1.00 | **0.046** |
| Others | 19.5% | 1.01 | 0.60 – 1.70 | 0.965 | 0.99 | 0.58 – 1.68 | 0.963 |
| **Structural readiness** |  |  |  |  |  |  |  |
| Corticosteroid availability *versus* unavailability^6^ | 45.1% *versus* 18.9% | 2.72 | 2.43 – 3.04 | **<0.001** | 1.14 | 1.01 – 1.29 | **0.038** |
| Ultrasound availability *versus* unavailability^6^ | 60.9% *versus* 19.3% | 5.03 | 4.51 – 5.61 | **<0.001** | 1.27 | 1.11 – 1.46 | **0.001** |
| *Readiness tertile*^7^ |  |  |  |  |  |  |  |
| High | 48.0% | 7.89 | 6.64 – 9.38 | **<0.001** | 1.96 | 1.61 – 2.38 | **<0.001** |
| Middle | 21.9% | 2.35 | 1.96 – 2.81 | **<0.001** | 1.26 | 1.05 – 1.52 | **0.014** |
| Low | 14.6% | *ref* |  |  | *ref* |  |  |
| *Staffing*^8^ |  |  |  |  |  |  |  |
| At least one medical doctor *versus* no medical doctor^9^ | 56.5% *versus* 13.2% | 6.44 | 5.71 – 7.27 | **<0.001** | 1.45 | 1.23 – 1.70 | **<0.001** |
| At least one midwife *versus* no midwife^9^ | 38.8% *versus* 25.1% | 3.16 | 2.83 – 3.54 | **<0.001** | 1.23 | 1.08 – 1.40 | **0.002** |
| At least one specialist *versus* no specialist^9^ | 61.8% *versus* 23.4% | 4.44 | 3.97 – 4.98 | **<0.001** | 1.21 | 1.05 – 1.39 | **0.010** |
| **CEmONC signal functions**^10^ |  |  |  |  |  |  |  |
| Ever provide parenteral antibiotics *versus* never | 37.8% *versus* 6.1% | 7.10 | 5.62 – 8.97 | **<0.001** | 1.38 | 1.07 – 1.79 | **0.013** |
| Ever provide parenteral oxytocin *versus* never | 33.1% *versus* 5.5% | 9.29 | 6.00 – 14.37 | **<0.001** | 1.84 | 1.16 – 2.92 | **0.009** |
| Ever provide parenteral anticonvulsants *versus* never | 46.2% *versus* 11.5% | 5.38 | 4.72 – 6.13 | **<0.001** | 1.63 | 1.41 – 1.89 | **<0.001** |
| Ever perform assisted vaginal delivery *versus* never | 37.1% *versus* 12.6% | 3.47 | 2.93 – 4.11 | **<0.001** | 1.13 | 0.94 – 1.36 | 0.181 |
| Ever perform manual removal of placenta *versus* never | 38.2% *versus* 7.2% | 5.61 | 4.56 – 6.92 | **<0.001** | 1.98 | 1.58 – 2.48 | **<0.001** |
| Ever perform removal of retained products *versus* never | 39.8% *versus* 11.8% | 3.71 | 3.21 – 4.28 | **<0.001** | 1.40 | 1.20 – 1.64 | **<0.001** |
| Ever perform neonatal resuscitation *versus* never | 36.7% *versus* 4.4% | 9.18 | 6.85 – 12.29 | **<0.001** | 2.55 | 1.88 – 3.46 | **<0.001** |
| Ever perform Cesarean sections *versus* never | 63.5% *versus* 13.1% | 7.94 | 7.06 – 8.93 | **<0.001** | 1.78 | 1.46 – 2.16 | **<0.001** |
| Ever provide blood transfusion *versus* never | 63.8% *versus* 15.1% | 6.80 | 6.09 – 7.60 | **<0.001** | 1.41 | 1.18 – 1.68 | **<0.001** |
| **Random effects** |  |  |  |  |  |  |  |
| N of region |  |  |  |  | 106 |  |  |
| τ00 region |  |  |  |  | 0.12 |  |  |
| ICC^11^ |  |  |  |  | 0.07 |  |  |
| Observations (N) |  |  |  |  | 6012 |  |  |
| Marginal R^2^/Conditional R^2^ |  |  |  |  | 0.614/0.639 |  |  |

^1^ Recent ACS use was defined as using ACS within the past 3 months. For each country, the proportions were calculated considering facility sampling weight.

^2^ Risk ratios (RR) were derived from bivariate regressions that included each independent variable separately, country fixed effects, and sub-national divisions as random intercepts.

^3^ Adjusted risk ratios (aRR) were derived from mixed effect log binomial regression model that included all independent variables, country fixed effects, and sub-national regions as random intercepts.

^4^ Bangladesh was selected as the reference level because the directions of risk ratios for other countries remained the same, facilitating a clearer interpretation and understanding of the effects.

^5^ Rural is the reference level.

^6^ Unavailability is the reference level.

^7^ Readiness tertiles refer to country-specific readiness tertiles, calculated by the available numbers of equipment, diagnostics, medicines and commodities, and guidelines.

^8^ These binary variables indicate facilities having at least one medical doctor, midwife, or specialist, which were constructed based on the surveyed staff types for each SPA.

^9^ No medical doctor, no midwife, or no specialist is the reference level.

^10^ Facilities that never performed each CEmONC signal function were viewed as the reference level

^11^ Intraclass correlation coefficient
