# Supplementary material for: Factors affecting antenatal corticosteroid use in low- and middle-income countries: Facility characteristics, structural readiness, and past performance of CEmONC signal functions
Source: PLOS Glob Public Health. 2025 Aug 14;5(8):e0003989. doi: 10.1371/journal.pgph.0003989 (PMC12352826; doi:10.1371/journal.pgph.0003989)
Supplement: S6 Table — (DOCX) [file pgph.0003989.s006.docx]

**S6 Table.** Country-specific regressions of recent antenatal corticosteroid utilization^1^

|  | **Afghanistan**  (n=99) | | | **Bangladesh**  (n=810) | | | **Nepal**  (n=800) | | |
| --- | --- | --- | --- | --- | --- | --- | --- | --- | --- |
|  | **Adjusted relative risk (aRR)** | **95% CI** | **p-value** | **Adjusted relative risk (aRR)** | **95% CI** | **p-value** | **Adjusted relative risk (aRR)** | **95% CI** | **p-value** |
| **Facility characteristic** |  |  |  |  |  |  |  |  |  |
| *Location* |  |  |  |  |  |  |  |  |  |
| Urban | -^2^ | - | - | 1.12 | 1.05 – 1.20 | **0.001** | 1.02 | 0.98 – 1.06 | 0.351 |
| Rural | -^2^ | - | - | *ref* |  |  | *ref* |  |  |
| *Managing authority type* |  |  |  |  |  |  |  |  |  |
| Public | *ref* |  |  | *ref* |  |  | *ref* |  |  |
| Private for-profit | 0.93 | 0.83 – 1.04 | 0.216 | 1.03 | 0.97 – 1.09 | 0.293 | 0.89 | 0.82 – 0.96 | **0.004** |
| Private not-for-profit | 0.99 | 0.82 – 1.19 | 0.897 | 0.97 | 0.88 – 1.06 | 0.474 | 0.92 | 0.79 – 1.07 | 0.273 |
| Others |  |  |  | - |  |  | - |  |  |
| **Structural readiness** |  |  |  |  |  |  |  |  |  |
| Corticosteroid availability | 1.01 | 0.88 – 1.17 | 0.852 | 1.04 | 0.99 – 1.10 | 0.143 | 1.04 | 1.00 – 1.08 | **0.036** |
| Ultrasound availability | 0.87 | 0.79 – 0.96 | **0.005** | 1.00 | 0.95 – 1.06 | 0.924 | 1.01 | 0.96 – 1.07 | 0.628 |
| *Readiness tertile* |  |  |  |  |  |  |  |  |  |
| High | 1.05 | 0.92 – 1.19 | 0.488 | 0.95 | 0.81 – 1.12 | 0.540 | 0.97 | 0.89 – 1.05 | 0.466 |
| Middle | 0.97 | 0.85 – 1.10 | 0.606 | 0.92 | 0.79 – 1.08 | 0.322 | 0.94 | 0.87 – 1.02 | 0.152 |
| Low | *ref* |  |  | *ref* |  |  | *ref* |  |  |
| *Staffing* |  |  |  |  |  |  |  |  |  |
| At least one medical doctor | 1.03 | 0.94 – 1.13 | 0.551 | 1.04 | 0.98 – 1.11 | 0.219 | 1.01 | 0.94 – 1.09 | 0.692 |
| At least one midwife | 0.62 | 0.42 – 0.91 | 0.015 | 1.05 | 0.99 – 1.11 | 0.088 | 0.98 | 0.91 – 1.06 | 0.690 |
| At least one specialist | 1.02 | 0.90 – 1.16 | 0.723 | 1.03 | 0.96 – 1.10 | 0.473 | 1.02 | 0.94 – 1.11 | 0.608 |
| **CEmONC signal functions** |  |  |  |  |  |  |  |  |  |
| Provide parenteral antibiotics | 1.22 | 1.04 – 1.44 | **0.018** | 1.05 | 1.00 – 1.11 | 0.071 | 1.01 | 0.98 – 1.04 | 0.446 |
| Provide parenteral oxytocin | 0.94 | 0.83 – 1.07 | 0.343 | 1.01 | 0.96 – 1.07 | 0.661 | 0.97 | 0.92 – 1.02 | 0.205 |
| Provide parenteral anticonvulsants | 1.20 | 1.03 – 1.40 | **0.019** | 1.13 | 1.07 – 1.19 | **<0.001** | 1.07 | 1.02 – 1.15 | **0.007** |
| Perform assisted vaginal delivery | 0.90 | 0.77 – 1.05 | 0.195 | 1.01 | 0.97 – 1.05 | 0.719 | 1.07 | 1.01 – 1.14 | 0.026 |
| Perform manual removal of placenta | 1.08 | 0.83 – 1.40 | 0.560 | 1.05 | 1.00 – 1.11 | 0.043 | 0.99 | 0.95 – 1.02 | 0.441 |
| Perform removal of retained products | 1.14 | 0.74 – 1.77 | 0.550 | 1.09 | 1.04 – 1.15 | **<0.001** | 1.05 | 1.01 – 1.09 | **0.012** |
| Perform neonatal resuscitation | 1.29 | 1.10 – 1.51 | **0.002** | 1.06 | 1.01 – 1.11 | **0.013** | 1.03 | 0.99 – 1.06 | 0.098 |
| Perform Cesarean sections | 1.59 | 1.38 – 1.84 | **<0.001** | 1.02 | 0.95 – 1.10 | 0.607 | 1.23 | 1.08 – 1.41 | **0.002** |
| Provide blood transfusion | 0.91 | 0.82 – 1.01 | 0.066 | 1.07 | 1.01 – 1.14 | **0.026** | 1.07 | 0.96 – 1.20 | 0.234 |

^1^ Modified Poisson regressions (Poisson regression with a robust error variance) were used to obtain adjusted relative risks (aRR).

^2^ Location (urban versus rural) was removed from the model as most of the facilities in Afghanistan were urban.

**S6 Table.** Country-specific regressions of recent antenatal corticosteroid utilization (continued) ^1^

|  | **Haiti**  (n=361) | | | **DRC**  (n=1329) | | | **Ethiopia**  (n=640) | | |
| --- | --- | --- | --- | --- | --- | --- | --- | --- | --- |
|  | **Adjusted relative risk (aRR)** | **95% CI** | **p-value** | **Adjusted relative risk (aRR)** | **95% CI** | **p-value** | **Adjusted relative risk (aRR)** | **95% CI** | **p-value** |
| **Facility characteristic** |  |  |  |  |  |  |  |  |  |
| *Urban versus rural* |  |  |  |  |  |  |  |  |  |
| Urban | 1.02 | 0.95 – 1.09 | 0.653 | 1.01 | 0.96 – 1.05 | **0.794** | 1.05 | 0.99 – 1.10 | 0.104 |
| Rural | *ref* |  |  | *ref* |  |  | *ref* |  |  |
| *Managing authority type* |  |  |  |  |  |  |  |  |  |
| Public | *ref* |  |  | *ref* |  |  | *ref* |  |  |
| Private for-profit | 0.94 | 0.87 – 1.02 | 0.152 | 0.94 | 0.89 – 1.00 | **0.036** | 0.92 | 0.86 – 0.99 | **0.020** |
| Private not-for-profit | 1.05 | 0.97 – 1.14 | 0.198 | 0.96 | 0.93 – 1.00 | 0.039 | 0.99 | 0.90 – 1.09 | 0.872 |
| Others | 1.02 | 0.95 – 1.10 | 0.595 | - |  |  | - |  |  |
| **Structural readiness** |  |  |  |  |  |  |  |  |  |
| Corticosteroid availability | 1.05 | 0.98 – 1.13 | 0.168 | 1.02 | 0.98 – 1.06 | 0.400 | 0.98 | 0.94 – 1.02 | 0.410 |
| Ultrasound availability | 1.07 | 0.98 – 1.17 | 0.144 | 1.08 | 1.03 – 1.13 | **0.002** | 0.99 | 0.93 – 1.05 | 0.726 |
| *Readiness tertile* |  |  |  |  |  |  |  |  |  |
| High | 1.16 | 1.00 – 1.34 | 0.044 | 1.18 | 1.12 – 1.24 | **<0.001** | 1.11 | 1.03 – 1.20 | **0.008** |
| Middle | 1.11 | 0.96 – 1.29 | 0.158 | 1.05 | 1.00 – 1.10 | **0.037** | 1.11 | 1.04 – 1.19 | **0.003** |
| Low | *ref* |  |  | *ref* |  |  | *ref* |  |  |
| *Staffing*^5^ |  |  |  |  |  |  |  |  |  |
| At least one medical doctor | 0.96 | 0.89 – 1.04 | 0.357 | 1.09 | 1.02 – 1.18 | **0.017** | 1.05 | 0.97 – 1.14 | 0.259 |
| At least one midwife | 1.01 | 0.93 – 1.09 | 0.857 | 1.03 | 0.98 – 1.07 | 0.220 | 1.05 | 1.00 – 1.10 | 0.053 |
| At least one specialist | 1.00 | 0.93 – 1.08 | 0.937 | 1.05 | 0.99 – 1.11 | 0.093 | 1.01 | 0.96 – 1.06 | 0.635 |
| **CEmONC signal functions** |  |  |  |  |  |  |  |  |  |
| Provide parenteral antibiotics | 0.95 | 0.87 – 1.03 | 0.222 | 1.02 | 0.97 – 1.06 | 0.528 | 0.97 | 0.84 – 1.11 | 0.623 |
| Provide parenteral oxytocin | 0.99 | 0.89 – 1.10 | 0.835 | 1.03 | 0.94 – 1.13 | 0.492 | 1.09 | 0.91 – 1.31 | 0.353 |
| Provide parenteral anticonvulsants | 1.13 | 1.06 – 1.22 | **<0.001** | 1.06 | 1.02 – 1.10 | **0.004** | 1.15 | 1.07 – 1.25 | **<0.001** |
| Perform assisted vaginal delivery | 0.99 | 0.93 – 1.06 | 0.761 | 0.94 | 0.90 – 0.99 | **0.020** | 0.97 | 0.84 – 1.11 | 0.627 |
| Perform manual removal of placenta | 1.05 | 0.98 – 1.12 | 0.180 | 1.07 | 1.02 – 1.13 | **0.010** | 1.10 | 0.98 -1.23 | 0.102 |
| Perform removal of retained products | 1.05 | 0.98 – 1.12 | 0.140 | 1.02 | 0.98 – 1.06 | 0.255 | 1.05 | 0.95 – 1.15 | 0.356 |
| Perform neonatal resuscitation | 1.06 | 1.00 – 1.12 | 0.068 | 1.11 | 1.05 – 1.16 | **<0.001** | 1.07 | 0.94 – 1.21 | 0.327 |
| Perform Cesarean sections | 1.19 | 1.01 – 1.40 | **0.036** | 1.04 | 0.97 – 1.12 | 0.255 | 1.25 | 1.12 – 1.40 | **<0.001** |
| Provide blood transfusion | 1.09 | 0.95 – 1.25 | 0.228 | 1.06 | 1.00 – 1.12 | **0.041** | 1.00 | 0.90 – 1.10 | 0.941 |

^1^ Modified Poisson regressions (Poisson regression with a robust error variance) were used to obtain adjusted relative risks (aRR).

**S6 Table.** Country-specific regressions of recent antenatal corticosteroid utilization (continued)^1^

|  | **Malawi**  (n=533) | | | **Senegal**  (n=591) | | | **Tanzania**  (n=948) | | |
| --- | --- | --- | --- | --- | --- | --- | --- | --- | --- |
|  | **Adjusted relative risk (aRR)** | **95% CI** | **p-value** | **Adjusted relative risk (aRR)** | **95% CI** | **p-value** | **Adjusted relative risk (aRR)** | **95% CI** | **p-value** |
| **Facility characteristic** |  |  |  |  |  |  |  |  |  |
| *Urban versus rural* |  |  |  |  |  |  |  |  |  |
| Urban | 1.02 | 0.95 – 1.10 | **0.575** | 0.90 | 0.82 – 1.00 | 0.052 | 0.98 | 0.93 – 1.03 | 0.427 |
| Rural | *ref* |  |  | *ref* |  |  | *ref* |  |  |
| *Managing authority type* |  |  |  |  |  |  |  |  |  |
| Public | *ref* |  |  | *ref* |  |  | *ref* |  |  |
| Private for-profit | 0.93 | 0.82 – 1.05 | 0.242 | 1.06 | 0.96 – 1.18 | 0.229 | 1.02 | 0.95 – 1.11 | 0.563 |
| Private not for-profit | 0.99 | 0.93 – 1.05 | 0.667 | 0.94 | 0.83 – 1.06 | 0.334 | 0.96 | 0.91 – 1.02 | 0.167 |
| Others | 1.02 | 0.87 – 1.20 | 0.819 | - |  |  | 0.93 | 0.77 – 1.12 | 0.442 |
| **Structural readiness** |  |  |  |  |  |  |  |  |  |
| Corticosteroid availability | 1.06 | 0.97 – 1.16 | 0.220 | 1.00 | 0.96 – 1.05 | 0.904 | 1.01 | 0.94 – 1.08 | 0.808 |
| Ultrasound availability | 1.18 | 1.02 – 1.37 | **0.028** | -^2^ |  |  | 1.14 | 1.05 – 1.23 | **0.003** |
| *Readiness tertile^4^* |  |  |  |  |  |  |  |  |  |
| High | 1.11 | 1.03 – 1.20 | **0.005** | 1.14 | 1.05 – 1.23 | **0.001** | 1.09 | 1.03 – 1.15 | **0.003** |
| Middle | 1.01 | 0.95 – 1.07 | 0.802 | 0.99 | 0.93 – 1.06 | 0.881 | 1.00 | 0.98 – 1.03 | 0.755 |
| Low | *ref* |  |  | *ref* |  |  | *ref* |  |  |
| *Staffing*^5^ |  |  |  |  |  |  |  |  |  |
| At least one medical doctor | 1.21 | 1.05 – 1.39 | **0.009** | 1.08 | 1.00 – 1.17 | 0.051 | 1.04 | 0.96 – 1.12 | 0.365 |
| At least one midwife | 1.02 | 0.92 – 1.13 | 0.721 | 0.99 | 0.93 – 1.06 | 0.834 | 1.01 | 0.98 – 1.04 | 0.484 |
| At least one specialist | 1.01 | 0.90 – 1.12 | 0.915 | 1.13 | 1.02 – 1.26 | **0.020** | 1.01 | 0.94 – 1.09 | 0.786 |
| **CEmONC signal functions** |  |  |  |  |  |  |  |  |  |
| Provide parenteral antibiotics | 1.07 | 1.00 – 1.15 | 0.055 | 1.00 | 0.92 – 1.09 | 0.951 | 1.02 | 0.99 – 1.05 | 0.252 |
| Provide parenteral oxytocin | 0.88 | 0.67 – 1.16 | 0.372 | 1.08 | 1.01 – 1.15 | 0.033 | 1.01 | 0.97 – 1.06 | 0.598 |
| Provide parenteral anticonvulsants | 0.94 | 0.87 – 1.01 | 0.110 | 1.12 | 1.06 – 1.18 | **<0.001** | 0.98 | 0.94 – 1.02 | 0.327 |
| Perform assisted vaginal delivery | 1.01 | 0.96 – 1.07 | 0.629 | 1.05 | 0.88 – 1.26 | 0.554 | 1.01 | 0.98 – 1.05 | 0.473 |
| Perform manual removal of placenta | 1.07 | 1.01 – 1.14 | **0.019** | 1.12 | 1.07 – 1.18 | **<0.001** | 1.06 | **1.03 – 1.09** | **<0.001** |
| Perform removal of retained products | 1.01 | 0.96 – 1.07 | 0.605 | 1.01 | 0.94 – 1.09 | 0.759 | 1.01 | 0.97 – 1.04 | 0.678 |
| Perform neonatal resuscitation | 1.15 | 1.00 – 1.31 | **0.044** | 1.09 | 1.01 – 1.17 | **0.023** | 1.00 | 0.97 – 1.03 | 0.868 |
| Perform Cesarean sections | 1.07 | 0.90 – 1.27 | 0.466 | 1.05 | 0.93 – 1.20 | 0.409 | 1.05 | 0.95 – 1.17 | 0.338 |
| Provide blood transfusion | 0.96 | 0.81 – 1.12 | 0.588 | 1.04 | 0.92 – 1.17 | 0.580 | 1.11 | 1.00 – 1.24 | 0.057 |

^1^ Modified Poisson regressions (Poisson regression with a robust error variance) were used to obtain adjusted relative risks (aRR).

^2^ Ultrasound was removed from the model for Senegal because no facilities in the sample had ultrasound available.
