# Supplementary material for: Correction: Factors affecting antenatal corticosteroid use in low- and middle-income countries: Facility characteristics, structural readiness, and past performance of CEmONC signal functions
Source: PLOS Glob Public Health. 2026 May 12;6(5):e0006470. doi: 10.1371/journal.pgph.0006470 (PMC13166958; doi:10.1371/journal.pgph.0006470)
Supplement: S5 Table — (DOCX) [file pgph.0006470.s001.docx]

**S5 Table.** Mixed effect **logistic** regression models of recent antenatal corticosteroid utilization among eight countries (excluding Afghanistan)

|  | **Proportion of facilities with recent ACS use (%)**^1^ | **Bivariate regrssion**^2^ | | | **Multivariate regression**^3^ | | |
| --- | --- | --- | --- | --- | --- | --- | --- |
|  |  | **odds ratio**  **(OR)** | **95% CI** | **p-value** | **Adjusted**  **odds ratio (aOR)** | **95% CI** | **p-value** |
| **Country** |  |  |  |  |  |  |  |
| Nepal 2021 | 8.2% | **0.39** | **0.22 – 0.69** | 0.001 | **0.19** | **0.10 – 0.36** | <0.001 |
| Haiti 2017-2018 | 23.6% | **0.53** | **0.30 – 0.93** | **0.026** | **0.17** | **0.09 – 0.34** | <0.001 |
| DRC 2017-2018 | 22.2% | **1.07** | **0.69 – 1.67** | **0.758** | **0.71** | **0.42 – 1.21** | **0.208** |
| Ethiopia 2021-2022 | 27.4% | **1.78** | **1.06 – 3.00** | **0.030** | **0.85** | **0.46 – 1.55** | **0.588** |
| Malawi 2013-2014 | 21.5% | **0.37** | **0.20 – 0.69** | **0.002** | **0.42** | **0.20 – 0.86** | **0.018** |
| Senegal 2018 and 2019 | 23.4% | **0.61** | **0.37 – 1.00** | **0.052** | **0.80** | **0.44 – 1.46** | **0.467** |
| Tanzania 2014-2015 | 4.0% | **0.29** | **0.19 – 0.47** | <0.001 | **0.14** | **0.08 – 0.24** | <0.001 |
| Bangladesh 2017-2018^4^ | 23.2% | *ref* |  |  | *ref* |  |  |
| **Facility characteristics** |  |  |  |  |  |  |  |
| *Location* |  |  |  |  |  |  |  |
| Urban *versus* rural^5^ | 44.5% *versus* 21.1% | **4.62** | **3.99 – 5.36** | **<0.001** | 1.33 | 1.09 – 1.61 | 0.004 |
| *Managing authority type* |  |  |  |  |  |  |  |
| Public | 28.0% | *ref* |  |  | *Ref* |  |  |
| Private for-profit | 43.0% | **1.84** | **1.52 – 2.21** | **<0.001** | 0.71 | 0.55 – 0.92 | **0.008** |
| Private not-for-profit/faith or mission-based | 37.8% | **1.70** | **1.43 – 2.03** | **<0.001** | 0.80 | 0.64 – 1.00 | **0.047** |
| Others | 19.5% | **1.05** | **0.59 – 1.89** | **0.865** | 0.98 | 0.50 – 1.92 | **0.947** |
| **Structural readiness** |  |  |  |  |  |  |  |
| Corticosteroid availability *versus* unavailability^6^ | 45.1% *versus* 18.9% | **3.46** | **3.02 – 3.97** | **<0.001** | **1.21** | **1.01 – 1.44** | **0.034** |
| Ultrasound availability *versus* unavailability^6^ | 60.9% *versus* 19.3% | **8.90** | **7.63 – 10.37** | **<0.001** | **1.45** | **1.17 – 1.79** | 0.001 |
| *Readiness tertile*^7^ |  |  |  |  |  |  |  |
| High | 48.0% | **15.89** | **12.72 –19.84** | **<0.001** | **2.63** | **2.02 – 3.42** | <0.001 |
| Middle | 21.9% | **2.83** | **2.29 – 3.49** | **<0.001** | **1.32** | **1.04 – 1.68** | **0.021** |
| Low | 14.6% | *ref* |  |  | *ref* |  |  |
| *Staffing*^8^ |  |  |  |  |  |  |  |
| At least one medical doctor *versus* no medical doctor^9^ | 56.5% *versus* 13.2% | **10.25** | **8.81 – 11.92** | **<0.001** | **1.52** | **1.23 – 1.89** | <0.001 |
| At least one midwife *versus* no midwife^9^ | 38.8% *versus* 25.1% | **5.16** | **4.36 – 6.09** | **<0.001** | **1.36** | **1.11 – 1.67** | **0.003** |
| At least one specialist *versus* no specialist^9^ | 61.8% *versus* 23.4% | **8.02** | **6.77 – 9.51** | **<0.001** | **1.42** | **1.13 – 1.79** | **0.003** |
| **CEmONC signal functions**^10^ |  |  |  |  |  |  |  |
| Ever provide parenteral antibiotics *versus* never | 37.8% *versus* 6.1% | **9.25** | **7.20 – 11.88** | **<0.001** | **1.39** | **1.03 – 1.89** | **0.031** |
| Ever provide parenteral oxytocin *versus* never | 33.1% *versus* 5.5% | **12.47** | **7.88 – 19.72** | **<0.001** | **1.88** | **1.07 – 3.31** | **0.028** |
| Ever provide parenteral anticonvulsants *versus* never | 46.2% *versus* 11.5% | **7.80** | **6.67 – 9.12** | **<0.001** | **1.87** | **1.54 – 2.27** | <0.001 |
| Ever perform assisted vaginal delivery *versus* never | 37.1% *versus* 12.6% | **4.41** | **3.63 – 5.35** | **<0.001** | **1.15** | **0.90 – 1.46** | **0.271** |
| Ever perform manual removal of placenta *versus* never | 38.2% *versus* 7.2% | **7.07** | **5.65 – 8.85** | **<0.001** | **2.22** | **1.69 – 2.91** | <0.001 |
| Ever perform removal of retained products *versus* never | 39.8% *versus* 11.8% | **4.78** | **4.06 – 5.63** | **<0.001** | **1.53** | **1.24 – 1.88** | <0.001 |
| Ever perform neonatal resuscitation *versus* never | 36.7% *versus* 4.4% | **11.96** | **8.81 – 16.22** | **<0.001** | **3.06** | **2.16 – 4.34** | <0.001 |
| Ever perform Cesarean sections *versus* never | 63.5% *versus* 13.1% | **14.92** | **12.73 – 17.48** | **<0.001** | **2.17** | **1.66 – 2.85** | <0.001 |
| Ever provide blood transfusion *versus* never | 63.8% *versus* 15.1% | **12.49** | **10.73 – 14.54** | **<0.001** | **1.66** | **1.28 – 2.14** | <0.001 |
| **Random effects** |  |  |  |  |  |  |  |
| N of region |  |  |  |  | 106 |  |  |
| τ00 region |  |  |  |  | **0.27** |  |  |
| ICC^11^ |  |  |  |  | **0.08** |  |  |
| Observations (N) |  |  |  |  | 6012 |  |  |
| Marginal R^2^/Conditional R^2^ |  |  |  |  | **0.578/0.610** |  |  |

^1^ Recent ACS use was defined as using ACS within the past 3 months. For each country, the proportions were calculated considering facility sampling weight.

^2^ Odds ratios (OR) were derived from bivariate regressions that included each independent variable separately, country fixed effects, and sub-national divisions as random intercepts.

^3^ Adjusted odds ratios (aOR) were derived from mixed effect logistic regression model that included all independent variables, country fixed effects, and sub-national regions as random intercepts.

^4^ Bangladesh was selected as the reference level because the directions of **odds** ratios for other countries remained the same, facilitating a clearer interpretation and understanding of the effects.

^5^ Rural is the reference level.

^6^ Unavailability is the reference level.

^7^ Readiness tertiles refer to country-specific readiness tertiles, calculated by the available numbers of equipment, diagnostics, medicines and commodities, and guidelines.

^8^ These binary variables indicate facilities having at least one medical doctor, midwife, or specialist, which were constructed based on the surveyed staff types for each SPA.

^9^ No medical doctor, no midwife, or no specialist is the reference level.

^10^ Facilities that never performed each CEmONC signal function were viewed as the reference level

^11^ Intraclass correlation coefficient
